# African American Females Are Less Metabolically Flexible Compared with Caucasian American Females following a Single High-Fat Meal: A Pilot Study

**DOI:** 10.3390/ijerph191912913

**Published:** 2022-10-09

**Authors:** Alyssa A. Olenick, Regis C. Pearson, Nuha Shaker, Maire M. Blankenship, Rachel A. Tinius, Lee J. Winchester, Evie Oregon, Jill M. Maples

**Affiliations:** 1 Division of Endocrinology, Metabolism & Diabetes, University of Colorado Anschutz Medical Campus, Aurora, CO 80045, USA; 2 Department of Pathology and Lab Medicine, University of Kentucky, Lexington, KY 40536, USA; 3 School of Nursing and Allied Health, Western Kentucky University, Bowling Green, KY 42101, USA; 4 School of Kinesiology, Recreation, and Sport, Western Kentucky University, Bowling Green, KY 42101, USA; 5 Department of Kinesiology, College of Education, The University of Alabama, Tuscaloosa, AL 35487, USA; 6 Department of Obstetrics and Gynecology, University of Tennessee Graduate School of Medicine, Knoxville, TN 37920, USA

**Keywords:** metabolic flexibility, African American, inflammation, high-fat meal

## Abstract

The relationship between metabolic flexibility (MF) and components of metabolic disease has not been well-studied among African American (AA) females and may play a role in the higher incidence of chronic disease among them compared with Caucasian American (CA) females. This pilot study aimed to compare the metabolic response of AA and CA females after a high-fat meal. Eleven AA (25.6 (5.6) y, 27.2 (6.0) kg/m^2^, 27.5 (9.7) % body fat) and twelve CA (26.5 (1.5) y, 25.7 (5.3) kg/m^2^, 25.0 (7.4) % body fat) women free of cardiovascular and metabolic disease and underwent a high-fat meal challenge (55.9% fat). Lipid oxidation, insulin, glucose, and interleukin (IL)-8 were measured fasted, 2 and 4 h postprandial. AA females had a significantly lower increase in lipid oxidation from baseline to 2 h postprandial (*p* = 0.022), and trended lower at 4 h postprandial (*p* = 0.081) compared with CA females, indicating worse MF. No group differences in insulin, glucose or HOMA-IR were detected. IL-8 was significantly higher in AA females compared with CA females at 2 and 4 h postprandial (*p* = 0.016 and *p* = 0.015, respectively). These findings provide evidence of metabolic and inflammatory disparities among AA females compared with CA females that could serve as a predictor of chronic disease in individuals with a disproportionately higher risk of development.

## 1. Introduction

Metabolic disease rates are higher among African American (AA) females compared with Caucasian American (CA) females [1,2,3]. Metabolic disease is classified by hallmark characteristics such as glucose and lipid dysregulation, elevated insulin, and inflammation. Metabolic flexibility is the ability of an organism to properly adjust substrate utilization in response to dietary intake or circulating substrates [4,5]. A lack of metabolic flexibility has been a proposed mechanism contributing to metabolic disease, including insulin resistance and systemic inflammation [4,5]. In addition, poor metabolic flexibility may play a role in the progression of metabolic syndrome and the improper utilization of glucose and lipids [6,7], ultimately contributing to higher incidences of metabolic disease among AA females [8,9].

However, the relationship between metabolic flexibility and components of metabolic disease has not been well-studied among AA females. One study found that AA females fail to significantly suppress lipid oxidation during a pancreatic–euglycemic clamp, despite doubling their fasting plasma insulin, and are unable to increase lipid oxidation or decrease carbohydrate oxidation in response to epinephrine infusion, as comparable to CA females [10]. Interestingly, and counter to previous work [10], a different study found that, when controlling for insulin sensitivity and diabetes status, AA individuals have a greater metabolic flexibility response after a hyperinsulinemic–euglycemic clamp compared with CA individuals [11]. However, this same group found that insulin sensitivity was a significant predictor of metabolic flexibility and that insulin sensitivity explained 48% of metabolic flexibility. More work is needed to understand the potential impact of race on metabolic flexibility among women.

There is a discrepancy in understanding the contributing factors of metabolic flexibility between different races, specifically among AA females, in response to real-world meal challenges. Postprandial lipid oxidation, insulin, and inflammatory response to a high-fat meal may be associated with impaired metabolic flexibility [9]; however, this has not been studied. Understanding how females of different races respond to a high-fat meal is important in further exploring the potential mechanisms behind the prevalence of diseases. With an increasing total time spent in a postprandial state, examining the metabolic and inflammatory response to a high-fat meal provides a real-world interpretation of possible racial differences in females. Therefore, the purpose of this pilot study was to compare metabolic flexibility, insulin response, and inflammation between AA and CA females after a high-fat meal to better understand racial metabolic differences that could be contributing to disproportionate rates of chronic disease. We hypothesize that AA females will show blunted postprandial metabolic flexibility and greater postprandial insulin and inflammatory response compared with CA females.

## 2. Materials and Methods

Ethical approval. The study was approved by the Western Kentucky University Institutional Review Board (IRB #17-021), with written informed consent being obtained prior to any experimental procedures. The study conformed to the standards set by the Declaration of Helsinki, except for registration in a database. Participants were recruited from similar geographic and socioeconomic regions.

Human participants. Twenty-eight females were recruited via flyers and word-of-mouth advertising. Five participants were excluded from the final analysis due to incomplete study completion (n = 3) or not meeting study criteria (n = 2). Twenty-three participants completed and were included in the final analysis. All participants self-identified as either AA (n = 11) or CA (n = 12) females and were between 20 and 39 years old. The sample size of recruitment was a priori set based off previous studies examining postprandial fat oxidation with similar techniques to ours [10,12]. Post hoc power analysis (G*Power) with a total sample size of 23 participants and an alpha level of 0.05 revealed a power of 0.3 for the primary outcome of postprandial fat oxidation. Participants were free from overt disease, nonsmokers, and denied use of illegal drugs or taking medications known to alter metabolism (e.g., corticosteroids and anti-psychotics known to alter insulin resistance/metabolic profiles). All participants were following a standard diet and did not report following any specific diet that may alter metabolism such as the ketogenic diet or a low-fat high-carb diet. All participants were matched between groups for age and BMI to keep groups homogenous and reduce differences between AA and CA women. All women were recruited locally from the Bowling Green, KY area, and were of similar socioeconomic background to reduce bias and differences between groups due to demographic or social influences.

Subject involvement. Participants wore a hip-based accelerometer (ActiGraph wGT3X-BT, ActiGraph, Pensacola, FL, USA) for a week prior to testing to make sure physical activity status was not different between groups. Participants were given written instructions for consuming a meal standardized to their body weight the night before the study visit, as previously described [13]. All data were collected on a single testing day. All participants arrived in the morning for testing between 07:00 and 08:00 am for all testing sessions after an overnight fast (~10–12 h). Upon arrival, participants’ weight, height, fasting blood glucose (via handheld glucometer, OneTouch UltraMini, LifeScan, Inc., Milpitas, CA, USA) and blood pressure were taken. A baseline blood draw was obtained via butterfly angiocatheter in the antecubital region of the arm or the posterior surface of the hand and flushed with saline to maintain patency. Body fat percentage was determined via seven-site skinfold thickness with a caliper (Lange Skinfold Calipers, Beta Technology, Santa Cruz, CA, USA). Skinfold sizes were entered into a standardized equation that accounts for age to calculate body fat percentage and controls for race (Equations (1) and (2)) [14].

Caucasian American Women: %BFDXA = 22.044(logSF) + 0.053(age) + 0.179(weight) − 0.155(height) + 0.156(waist) − 13.093(1)

African American Women: %BFDXA = 20.867(logSF) + 0.052(age) + 0.140(weight) − 0.152(height) + 0.149(waist) − 8.227(2)

After baseline measures were obtained (~20–25 min), baseline fasting indirect calorimetry was performed. Expired oxygen and carbon dioxide were analyzed for 15 min using the TrueOne Canopy Option and TrueOne Metabolic Cart (TrueOne 2400, Parvomedics, Sandy, UT, USA). Lipid oxidation rates were calculated by the Frayn equation [15]. After the baseline blood draw, participants consumed a standardized high-fat meal, similar in composition to previous studies [12,16,17]. The high-fat meal consisted of a standardized smoothie from Smoothie King© that was prepared specifically for the study. Each participant consumed the same smoothie, which had 1062 total kilocalories, of which 66 g were from fat (55.93% kcal), 78 g from carbohydrates (29.38% kcal), and 39 g from protein (14.69% kcal). Participants were instructed to finish the shake within <10 min, and the timer was started as soon as they began drinking the smoothie. Additional indirect calorimetry measures and blood draws were taken at 2 and 4 h postprandial. Participants rested in a seated or supine position throughout the 4 h postprandial period, only getting up to use the restroom. Blood samples were immediately centrifuged for 10 min at 2200–2500 RPM. Plasma was separated and stored at −80 °C until final analyses were performed.

Whole blood quantification. Blood draws were used to analyze glucose, insulin, and interleukin (IL)-8. Whole blood samples for glucose were analyzed immediately with an automated glucose analyzer (OneTouch, Ultra 2, LifeScan, Inc.). Insulin and IL-8 were analyzed via standardized assays following the manufacturers’ instructions and ran in duplicate. Insulin and glucose levels assessed at baseline, 2 and 4 h postprandial were used to calculate the homeostatic model assessment—insulin resistance (HOMA-IR) and Matsuda’s Insulin Sensitivity Index (ISI) [18,19]. IL-8 concentrations were determined by ELISA (R&D Systems, Minneapolis, MN, USA). The inter-assay coefficient of variation was 3.8% with an intra-assay coefficient of variation of 6.7% for IL-8 samples. Insulin concentrations were determined by ELISA (Diazym, Poway, CA, USA). The inter-assay coefficient of variation was 1.2% with an intra-assay coefficient of variation of 1.6% for insulin samples.

Statistical analysis. To reduce bias in the analysis between groups, all data were processed and analyzed in the same exact way. Normality of the distribution for each variable was tested using Kolmogorov–Smirnov tests. Data used in the analyses met assumptions of sphericity and homogeneity of variance. Participants with missing or incomplete data were left out of the final analysis. Independent sample *t*-tests were performed for all baseline data (AA vs. CA). To determine the impact that race may have on metabolic flexibility, and fasted and postprandial insulin, glucose and IL-8 concentrations, a repeated-measures ANOVA with emphasis on race (AA vs. CA) and time (pre- vs. post-meal) were performed with LSD post hoc analysis. Metabolic flexibility was defined as the percent change in lipid oxidation rate in response to the high-fat meal at 2 and 4 h postprandial (i.e., metabolic flexibility = (postprandial lipid oxidation—baseline lipid oxidation)/baseline lipid oxidation). Pearson’s correlation coefficient was used to determine correlations between percent changes in IL-8, insulin and lipid oxidation (e.g., metabolic flexibility) from baseline to 2 and 4 h postprandial. Statistical analyses were performed using SPSS Statistics Software v26 (SPSS, Chicago, IL, USA). Statistical significance was accepted at *p* ≤ 0.05. Data are reported as mean (SD).

## 3. Results

### 3.1. Subject Characteristics

A total of 11 AA and 12 CA females were recruited and completed the pilot study. There were no significant differences between groups in terms of anthropometrics, blood pressure, resting energy expenditure, body composition, or measures of insulin sensitivity (*p* > 0.05, Table 1). There were no differences between groups in average activity calorie expenditure (CA: 236.7 (157.4), AA: 259.8 (132.1) kcal/week; *p* = 0.373) or percent of total time spend undergoing sedentary (CA: 86.4 (6.9), AA: 85.4 (6.8) % total activity; *p* = 0.785), light (CA: 6.0 (3.7), AA: 7.2 (3.2) % total activity; *p* = 0.518), moderate (CA: 3.4 (1.6), AA: 3.1 (1.4) % total activity; *p* = 0.684), vigorous (CA: 0.4 (0.4), AA: 0.3 (0.4) % total activity; *p* = 0.623) or very vigorous (CA: 0.2 (0.3), AA: 0.1 (0.0) % total activity; *p* = 0.165) physical activity.

### 3.2. Lipid Oxidation and Metabolic Flexibility

**Figure 1:**
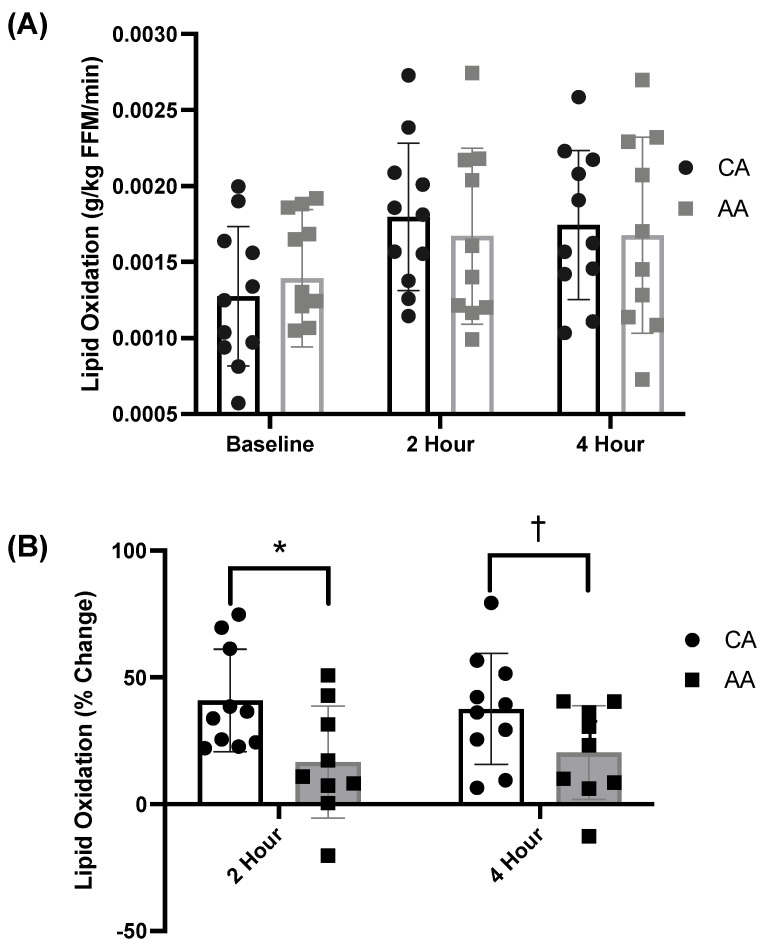
Baseline and postprandial fat oxidation and metabolic flexibly in response to a high-fat meal in African American and Caucasian American females. (**A**) Relative lipid oxidation (g/kg FFM/min; ANOVA: Time, *p* < 0.001, η_p_^2^ = 0.775; Group, *p* = 0.127, η_p_^2^ = 0.103; *n* = 23) response to a high-fat meal and (**B**) metabolic flexibility response to a high-fat meal (% change in lipid oxidation) at 2 and 4 h postprandial (*t*-test; * *p* = 0.022 and † *p* = 0.081). AA—African American; CA—Caucasian American.

There was a significant time effect of the meal on lipid oxidation (g/kg FFM/min; ANOVA: Time, *p* < 0.001, η_p_^2^ = 0.775, Figure 1A). There was no effect of race on lipid oxidation (g/kg FFM/min; ANOVA: Group, *p* = 0.127, η_p_^2^ = 0.103, Figure 1A). There were no differences in lipid oxidation (g/kg FFM/min) between AA and CA females at baseline or postprandially (*p* > 0.05, Figure 1A). However, CA females had a significantly greater increase in 2 h metabolic flexibility compared with AA females (*p* = 0.022, Figure 1B). Additionally, 4 h metabolic flexibility in CA females trended higher compared with AA females (*p* = 0.081, Figure 1B).

### 3.3. Plasma Insulin and Glucose

**Figure 2:**
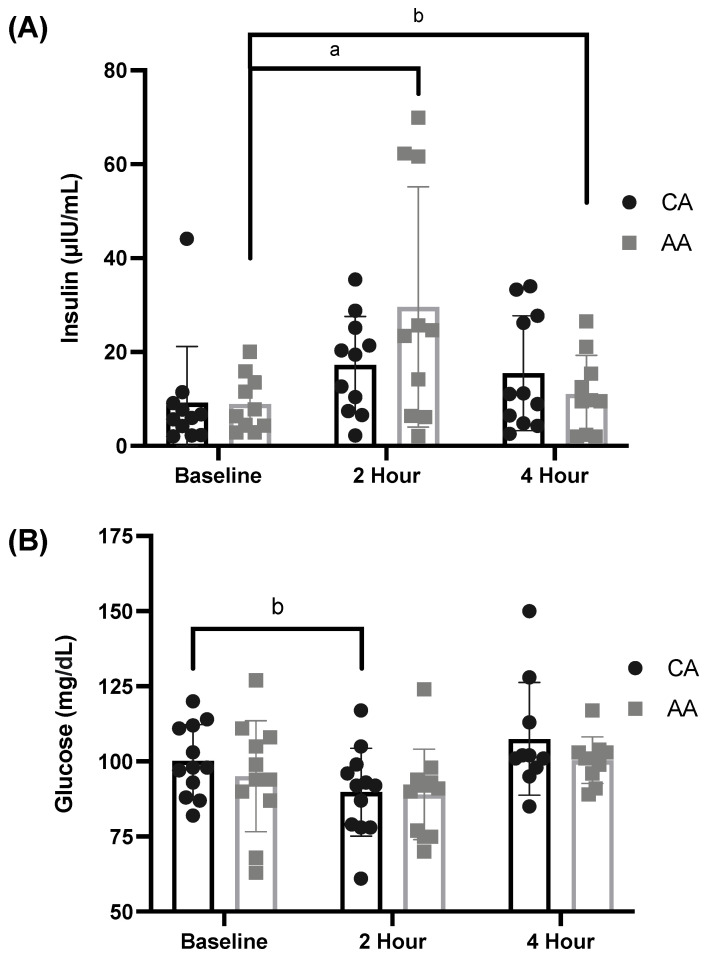
Baseline and postprandial insulin and glucose response to a high-fat meal in African American and Caucasian American females. (**A**) Insulin (μIU/mL) response to a high-fat meal (ANOVA: Time, *p* = 0.004, η_p_^2^ = 0.440; Group, *p* = 0.096, η_p_^2^ = 0.219; *n* = 23) and (**B**) glucose (mg/dL) response to a high-fat meal (ANOVA: Time, *p* < 0.001, η_p_^2^ = 0.633; Group, *p* = 0.875, η_p_^2^ = 0.016; *n* = 23). a *p* ≤ 0.05 compared to baseline for AA females and b *p* ≤ 0.05 compared to baseline for CA females. AA—African American; CA—Caucasian American.

There was a significant time effect of the meal on insulin (μIU/mL; ANOVA: Time, *p* = 0.004, η_p_^2^ = 0.440, Figure 2A) and glucose (mg/dL; ANOVA: Time, *p* < 0.001, η_p_^2^ = 0.633, Figure 2B). Insulin significantly increased from baseline to 2 h postprandial in AA females and increased from baseline to 4 h postprandial in CA females (*p* = 0.019 and *p* = 0.050, respectively, Figure 2A). There was a significant decrease in glucose from baseline to 2 h postprandial in CA females, but not in AA females (*p* = 0.038 and *p* = 0.129, respectively, Figure 2B).

There was no effect of race on insulin (μIU/mL; ANOVA: Group, *p* = 0.096, η_p_^2^ = 0.219, Figure 2A) or glucose (mg/dL; ANOVA: Group, *p* = 0.875, η_p_^2^ = 0.016, Figure 2B). Additionally, there was no interaction effect of time and race for insulin (ANOVA: Time × Group, *p* = 0.096, η_p_^2^ = 0.219, Figure 2A) or glucose (ANOVA: Time × Group, *p* = 0.875, η_p_^2^ = 0.016, Figure 2B).

### 3.4. Inflammation

**Figure 3:**
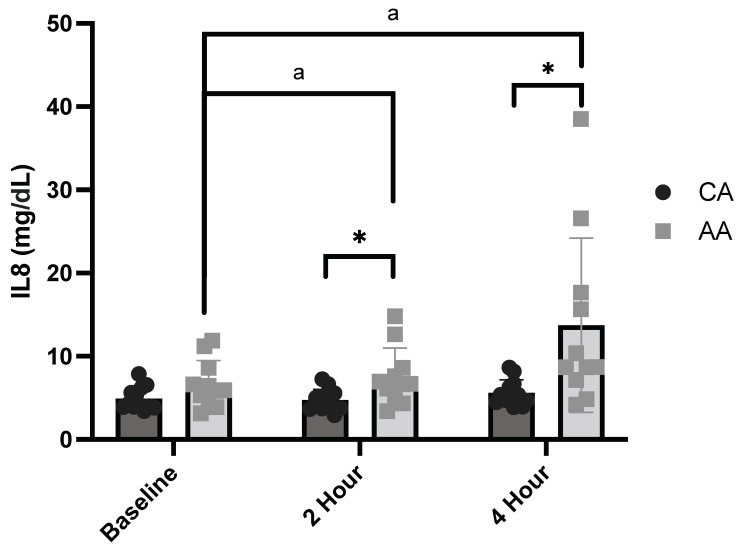
Baseline and postprandial IL-8 response to a high-fat meal in African American and Caucasian American females (ANOVA: Time, *p* = 0.039, η_p_^2^ = 0.277; Group, *p* = 0.050, η_p_^2^ = 0.259; *n* = 23). * *p* ≤ 0.05 between CA vs. AA females. * *p* ≤ 0.05 between AA and CA, a *p* ≤ 0.05 compared to baseline for AA females. AA—African American; CA—Caucasian American.

There was a significant time effect of the meal on IL-8 (mg/dL; ANOVA: Time, *p* = 0.039, η_p_^2^ = 0.277, Figure 3) and a significant time and race interaction for IL-8 (mg/dL; ANOVA: Time × Group, *p* = 0.050, η_p_^2^ = 0.259, Figure 3). IL-8 was significantly increased from baseline to 2 and 4 h postprandial in AA females (*p* = 0.023 and *p* = 0.003, respectively, Figure 3). Additionally, IL-8 was significantly higher in AA females compared with CA females at 2 and 4 h postprandial (*p* = 0.016 and *p* = 0.015, respectively, Figure 3), and non-significantly elevated at baseline (*p* = 0.074, Figure 3).

### 3.5. Exploratory Correlative Analysis between Metabolic Flexibility, Insulin, Glucose and IL-8 Concentrations

**Figure 4:**
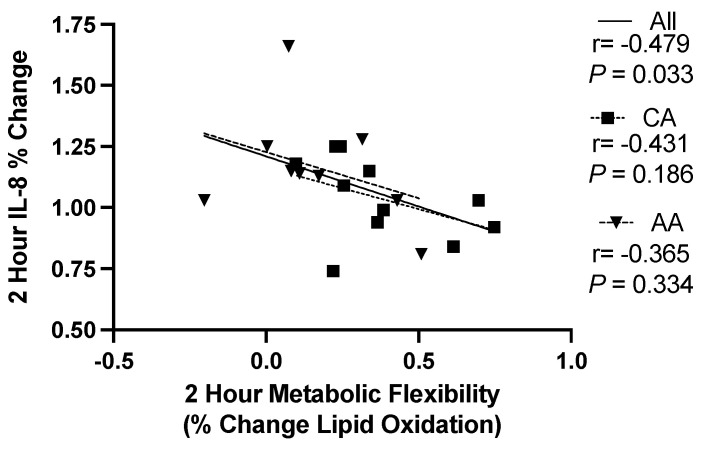
Exploratory correlative analysis between IL-8 percent change and metabolic flexibility from baseline to 2 h postprandial in response to a high-fat meal. AA—African American; CA—Caucasian American.

Overall, there was a significant relationship between the changes from baseline to 2 h postprandial for metabolic flexibility and percent change in IL-8 (r = −0.479, *p* = 0.033, Figure 4A), but not 2 h metabolic flexibility and percent change in insulin (r = 0.257, *p* = 0.287), 2 h metabolic flexibility and percent change in glucose (r = −0.180, *p* = 0.447), or 2 h postprandial percent change in insulin and IL-8 (r = −0.051, *p* = 0.821). When stratified by race, the relationship between 2 h metabolic flexibility and percent change in IL-8 was no longer significant (AA females *p* = 0.334 and CA females *p* = 0.186, Figure 4A).

Overall, there was no significant relationship between the changes from baseline to 4 h postprandial for metabolic flexibility and percent change in insulin (r = 0.116, *p* = 0.653), 4 h metabolic flexibility and percent change in glucose (r = 0.083, *p* = 0.720), 4 h metabolic flexibility and percent change in IL-8 (r = 0.083, *p* = 0.720), or percent change in insulin and IL-8 (r = −0.143, *p* = 0.526).

## 4. Discussion

The current pilot study examined metabolic flexibility, insulin response, and inflammation differences between AA and CA females after consuming a high-fat meal. AA females were less metabolically flexible than CA females in response to a high-fat meal. AA females increased their insulin responses at 2 h postprandial compared to baseline. Finally, AA females had a significantly greater postprandial IL-8 response compared with CA females. These findings provide evidence of metabolic and inflammatory disparities among AA females compared with their CA counterparts. These preliminary data suggest that race may play a meaningful role in postprandial metabolic flexibility and health in women, and should be further explored.

Individuals with type 2 diabetes and obesity demonstrate dampened metabolic flexibility [20,21,22], potentially driven by insulin resistance that encompasses defects in metabolic flexibility and lipid metabolism [5,6]. In the current study, AA females were less metabolically flexible after consuming a high-fat meal compared with CA females. Our present data established metabolic flexibility differences between AA and CA females in the absence of obesity (26.27 (8.25) % fat mass) and insulin resistance (HOMA-IR = 2.13 (1.33) [23]). It has been previously suggested that insulin sensitivity is one of the main contributors to metabolic flexibility; however, it is less clear if metabolic flexibility precedes or follows insulin resistance in African American women [11]. While no group differences for insulin resistance were found in the present study, African American women did have elevated insulin at 2 h postprandial, indicating that even in healthy females, insulin sensitivity may play a predominant role in metabolic flexibility. A blunted metabolic flexibility, as quantified by a change in postprandial lipid oxidation from fasting, may potentially serve as a precursor to the progression of metabolic disease.

While the role of insulin sensitivity and metabolic flexibility are tightly related in adults, racial differences in metabolic flexibility related to insulin sensitivity have yet to be thoroughly investigated. Herein, we found a significant increase in insulin 2 h postprandial in AA females. Elevated insulin may impair whole-body lipolysis [24], possibly contributing to the blunted lipid oxidation response in our AA females at 2 h postprandial. The impaired ability to upregulate lipid oxidation in the face of lipid oversupply may increase fat accumulation and contribute to insulin resistance [25]. Additionally, individuals with a family history of type 2 diabetes have impaired lipid oxidation rates after a high-fat meal compared with those without a family history [12].

Elevated systemic inflammation is related to metabolic syndrome [26], and a chronic high-fat [27] or high-calorie diet [28]. The inflammatory response to excess dietary intake may also extend to a single high-fat meal [26,27,29]. When chronically elevated, IL-8 plays an adverse pathophysiological role in cardiovascular disease development, recruiting immune cells to the site of vascular damage and promoting the increased production of other proinflammatory cytokines [30]. Relatively limited research has investigated the role of postprandial IL-8 concentrations after a high-fat meal. A 2017 systematic review concluded that postprandial IL-8 does not change transiently or robustly after a high-fat meal [27]. However, this conclusion was only limited to four studies. The current study found that AA females expressed significantly higher IL-8 concentrations postprandially compared to their CA counterparts. Furthermore, other data from our lab investigating peripheral blood mononuclear cell proteins and the gene expression of IL-8 within the same group of women found similar racial differences [13]. Interestingly, during our exploratory correlative analysis, we found a significant relationship between the change from baseline to 2 h postprandial for IL-8 and metabolic flexibility among all women. When separated by race, this significance was not found. We speculate there may be race and sex-specific changes in postprandial IL-8 concentrations that have not been detected in males or non-AA adults.

The connections between race, systemic inflammation, and metabolic flexibility, particularly in response to an acute high-fat meal, have not been fully elucidated. We speculate our data represent a circular relationship where elevated systemic inflammation and insulin response impede metabolic flexibility, which then, in return, further contributes to elevated inflammation and possibly insulin insensitivity. In otherwise healthy AA females, a decreased metabolic flexibility and higher inflammatory response in response to a single high-fat meal, compared with CA females, may represent precursors to long-term metabolic dysregulation. The concept of a cyclical interaction of race, inflammation, and metabolic flexibility necessitates further establishment through acute, chronic, and longitudinal investigations. Furthermore, differences in the lipolytic function of adipose and skeletal muscle tissue, as well as mitochondrial function and capacity, have been shown to influence lipid metabolism in African American vs. Caucasian women and may influence the differences seen here [31,32,33,34,35]. Future work should also continue to consider these factors in regulating metabolic flexibility and insulin resistance.

While our study controlled for general physical activity levels, it is worth noting that exercise training may improve metabolic flexibility in both African American and Caucasian women and may be a potential therapeutic target to rescue metabolic flexibility in these women [36,37]. Additionally, the current study only assessed 2 and 4 h postprandial time points, resulting in the immediate postprandial response (e.g., 30–60 min) being missed. This additional timepoint investigation could reveal specific insulin and lipid oxidation differences between groups [38,39]. A larger sample size may have also allowed further analyses of the relationship between race, insulin, inflammation, and lipid oxidation in the postprandial response, and offered stronger study power to draw conclusions from. Future work should aim to replicate our findings in a larger group of women. We also acknowledge the limitations of self-reporting on racial status on study outcomes. Lastly, we recognize the limitations of not having any further assessments on more inflammatory markers (IL-6, CRP, TNF-α, etc.) or mitochondrial phenotypes, which may influence substrate oxidation between African American and Caucasian women. Future studies should consider race, inflammatory markers and insulin response as strong predictors of metabolic flexibility, as well as therapeutic interventions such as exercise training for reversing metabolic inflexibility in African American women.

## 5. Conclusions

In summary, AA females had less metabolic flexibility and a higher inflammatory response after a single high-fat meal compared with CA females. Additionally, our findings support the potential for a measure of metabolic flexibility to serve as a potential predictor of future metabolic dysfunction in individuals with disproportionately higher risk for chronic disease [40]. Future investigations should consider race as a predominant contributor to metabolic flexibility and inflammatory response after a single high-fat meal. Lastly, work needs to be established concerning how to improve metabolic flexibility and systemic inflammation in at-risk populations. Exercise seems to be a potent stimulus to improve metabolic flexibility [41,42], systemic inflammation [43], and insulin resistance [44]. Additional work should be focused on examining differences in chronic exercise adaptations with specific respect to race.

## Figures and Tables

**Table 1:** Subject characteristics and insulin sensitivity values.

	CA Females	AA Females	t-Test *p*-Value
Age (y)	26.5 (1.5)	25.6 (5.6)	*p* = 0.714
Weight (kg)	70.1 (21.4)	75.1 (15.8)	*p* = 0.534
BMI (kg/m^2^)	25.7 (5.3)	27.2 (6.0)	*p* = 0.535
SBP (mmHg)	120.9 (11.8)	117.8 (12.3)	*p* = 0.575
DBP (mmHg)	75.5 (7.1)	72.1 (12.2)	*p* = 0.433
Fasted blood glucose (mg/dL)	100.2 (11.8)	95.0 (18.4)	*p* = 0.431
Waist circumference (cm)	33.5 (7.7)	33.1 (5.0)	*p* = 0.894
Hip circumference (cm)	39.1 (5.0)	40.2 (6.3)	*p* = 0.632
Waist to hip (ratio)	0.76 (0.1)	0.76 (0.07)	*p* = 0.787
Body fat percentage	25.0 (7.4)	27.5 (9.7)	*p* = 0.492
Lean mass (kg)	50.9 (11.2)	53.3 (6.1)	*p* = 0.537
REE (kcals/d)	1514.1 (272.4)	1450.8 (107.2)	*p* = 0.480
HOMA-IR	2.1 (2.6)	2.1 (1.3)	*p* = 0.935
Matsuda’s ISI from meal challenge	16.4 (16.2)	14.7 (11.8)	*p* = 0.796

Note: Data are reported as mean (SD). CA—Caucasian American; AA—African American; y—years; d—day; cm—centimeter; kg—kilogram; mmHg—millimeters of mercury; mg—milligram; dL—deciliters; REE—resting energy expenditure; kcals—kilocalories; ISI—insulin sensitivity index.

## Data Availability

The datasets generated during and/or analyzed during the current work are not publicly available but are available from the corresponding author on reasonable request.
